# The role of salicylic acid in plant flower development

**DOI:** 10.48130/FR-2022-0014

**Published:** 2022-10-31

**Authors:** Ying Luo, Meilan Liu, Jie Cao, Fuliang Cao, Lin Zhang

**Affiliations:** 1 Key Laboratory of Cultivation and Protection for Non-wood Forest Trees, Ministry of Education, Central South University of Forestry and Technology, Changsha 410004, Hunan, China; 2 Co-innovation Center for the Sustainable Forestry in Southern China, College of Forestry, Nanjing Forestry University, Nanjing 210037, Jiangsu, China

**Keywords:** Salicylic acid, Flowering, Stamen, Pollen, Ovary

## Abstract

Salicylic acid (SA), belonging to a family of naturally occurring phenolic compounds, is a crucial plant hormone involved in many biological processes, such as plant immunity, seed germination, root initiation, stomatal closure, and biotic/abiotic stress response. SA playing central roles in many metabolic processes have been extensively characterized. However, the function of SA in plant flower development has rarely been investigated. This paper reviews recent research advances on the roles of SA in flower development, including regulation of stamen development, flowering time, and ovarian development. This study provides multiple lines and levels of evidence substantiating that SA plays important roles in flower development, and suggests a pressing need to study the flower development-related functions of SA in both annual and perennial plants.

## Introduction

Salicylic acid (SA, 2-hydroxybenzoic acid) is one of the phenolics with hydroxyl group or derivatives thereof. It is usually present as free SA or as glycosylated and methylated compounds, whose contents and forms of presentation vary according to species, organs, developmental stages, and are influenced by environmental conditions and other factors^[1]^. SA is one of the most important phytohormones, and involved in various biological processes such as abiotic stress responses^[2]^, plant defense against pathogens^[3]^, and plant morphogenesis^[4−6]^, etc. Being a signaling molecule, SA responds to both internal and external signals and regulates downstream gene expression in various biological processes^[7]^.

As the current research of SA mainly focuses on the plant morphogenesis and stress responses, the functions of SA in flower development remain unexplored. Recently, an increasing number of studies revealed that SA is implicated in flower development of many plant species. For example, the concentration of SA increases sharply during the key differentiation stages of unisexual female flower formation^[4, 8]^, pollen germination^[9,10]^, pollen tube elongation^[11, 12]^, and post-fertilization ovary development^[13−15]^, suggesting that SA plays certain roles in these processes. However, the mechanism underlying these processes as well as whether SA works in other reproductive processes remain elusive and deserve to be investigated in depth in further studies. This review summarizes recent progress towards understanding the SA-involved flower development processes, including flowering, stamen development, pollen tube germination, pollen tube elongation, and ovary development.

## Review

### SA regulates stamen failure in female flowers

Flower differentiation is an important morphogenetic event in the process of sexual reproduction in higher plants. Abiotic stress-induced pollen abortion is always a consequence of their abnormal tapetum development^[10]^. It is evidenced that SA can alleviate damage under heat stress^[16]^, Cd-induced photosynthetic damage and programmed cell death (PCD)^[17]^. PCD also plays an important role in the developmental and stress-response processes of plants.

**Figure 1:**
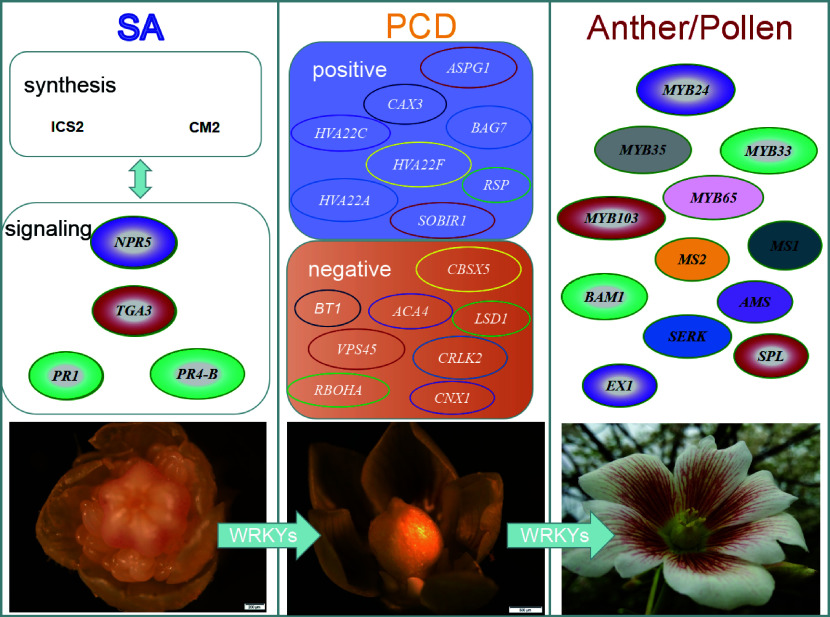
Hypothetical model for development of female flowers in *Vernicia fordii*. The SA triggers PCD in tapetum cells, leading to stamen abortion and female flower formation. Transcriptome analysis and co-expression networks suggest that SA accumulation may trigger PCD and inhibit stamen development in female flowers. ICS2: Isochorismate synthase 2; CM2: Chorimate mutase 2; *NPR5: Non-expressor of pathogenesis-related genes 5*;* TGA3*: *TGA1A-RELATED gene3*;* PR1*: *Pathogenesis- related 1*; *PR4-B*:* Pathogenesis-related 4-B*; *ASPG1*: *Aspartic protease in guard cell 1*; *CAX3*: *Vacuolar cation/proton exchanger 3*; *BAG7*: *BAG family molecular chaperone regulator 7*; *HVA22C*: *HVA22-like C*; *HVA22F*: *HVA22-like protein F*; *HVA22A*: *HVA22-like protein A*; *SOBIR1*: *Leucine-rich repeat receptor-like serine/threonine/tyrosine-protein kinase*; *RSP*: *CO(2)-response secreted protease*; *CBSX5*: *CBS domain-containing protein CBSX5*; *BT1*: *Adenine nucleotide transporter BT1*; *ACA4*: *Alpha carbonic anhydrase 4*; *LSD1*: *Lesion Simulating Disease 1*; *VPS45*: *Vacuolar protein sorting-associated protein 45 homolog*; *CNX1*: *Molybdopterin adenylyltransferase*; *RBOHA*: *Respiratory burst oxidase homolog protein A*; *CRLK2*: *Calcium/calmodulin-regulated receptor-like kinase 2;* *MYB24*: *MYB family transcription factor 24*; *MYB33* : *MYB family transcription factor 33*; *MYB35*: *MYB family transcription factor 35*; *MYB65* : *MYB family transcription factor 65*; *MYB103*: *MYB family transcription factor 103* ; *MS1*: *Male sterility 1*; *MS2*: *Fatty acyl-CoA reductase 2*; *BAM1*: *barely any meristem 1; AMS: aborted microspores*; *SERK*: *Somatic embryogenesis receptor-like kinase 1*; *SPL*: *sporocyteless*; *EX1*: *excess microsporocytes 1*.

SA-triggered PCD is found in tapetum cells, leading to stamen abortion during female flower formation in unisexual plants^[4]^. In unisexual plants such as *Vernicia fordii* and *Litsea cubeba*, some of the female flowers present bisexual primordia at early stages and switch to unisexual flowers at later stages due to the tapetum degeneration by PCD in stamens^[18]^. SA content was stage-specific during this process, being undetectable in the early stages, rising to a significantly high level when stamens started to degenerate, and falling to an extremely low level in the later stages when the stamens have disappeared^[4]^. However, in male flowers, SA remained at low levels across all developmental stages^[4]^. Transcriptome and co-expression network analysis showed that PCD- and SA-related genes were differentially expressed between developing male and female flowers, suggesting that SA accumulation may trigger PCD, inhibiting stamen development of female flowers in *V. fordii*^[4]^ (Fig. 1).

For dioecious plants, like *L. cubeba*, SA increased rapidly during the early developmental phase of female floral bud, reaching a peak at the stamen degeneration phase and decreasing gradually thereafter; whereas in female flowers, SA levels in male flowers decreased continuously^[5, 6]^. A bZIP family transcription factor of *L. cubeba*, *LcTGA10,* is a positive regulator for the SA signal transduction pathway. In the female flowers with degenerated stamens, the expression level of *LcTGA10* was significantly increased, and overexpressing *LcTGA10* showed a reduced number of stamens, smaller sepals, shorter styles, and reduced fertility^[5, 6, 19]^, suggesting that the accumulation of SA in pistillate flower may initiate the expression of PCD-related genes and trigger the degradation of stamens.

### SA regulates flowering time

Flowering is a key prerequisite stage for the irreversible transition from vegetative growth to reproductive growth in higher plants, where internal and external signals such as the developmental stage, phytohormones, light and temperature mediate the initiation of cell differentiation so that flowering occurs at the most appropriate time^[20]^. Flowering is precisely controlled by a complex signaling network, with the key regulators including flowering integrated gene of *FLOWERING LOCUS T* (*FT*), the transcription factor (TF) of *FLOWERING LOCUS D* (*FLD*), flower integration gene of *SUPPRESSOR OF OVEREXPRESSION OF CONSTANS 1* (*SOC1*), floral meristem identity gene of *APETALA 1* (*AP1*), and photoperiod pathway regulatory genes of *GIGANTEA* (*GI*) and *CONSTANS* (*CO*).

SA is found to be a crucial signal for flowering induction besides light and temperature. In tobacco callus, exogenous SA promoted the formation of flower buds^[21]^; in the long-day (LD) plant *Lemna gibba*, exogenous SA could appropriately shorten the light period required for flowering induction*,* and promote the transition from vegetative growth to reproductive growth in the absence of ${\text {NH}}_4^+ $^[22−24]^. Exogenous SA added in the liquid culture induces flowering in *L. gibba*^[25]^. Studies have shown that SA is required for the induction of flowering in some plants^[26, 27]^. For example, stress-induced SA accumulation accelerates flowering in *Arabidopsis* and *Lemna paucicostata*^[28, 29]^, while SA inhibits the floral repressor gene *FLOWERING LOCUS C* (*FLC*) and upregulates *FT* which then interacts with *FLD* via transcription and activates the expression of *SOC1* and *AP1* to promote flowering^[27]^. In SA-deficient *Arabidopsis*, the late-flowering phenotype was completely *FLC*-independent under LD conditions and partially *FLC*-dependent under short-day (SD) conditions. Expression of* CO* and* SOC1* in SA-deficient *Arabidopsis* (Arabidopsis with mutations in SA biosynthetic genes) differed under SD and LD conditions, suggesting that SA regulates flowering by interaction with the photoperiod-dependent pathway through a separate *CO*-independent pathway^[27]^. SA accelerates flowering by photoperiodic-independent and vernalization-independent pathways as well as autonomous and GA-dependent pathways^[30]^. The TF of *HAHB10* from *Helianthus annuus*, belongs to the HD-Zip II subfamily, promotes flowering by inducing flowering transition genes such as *FT*, *FRUITFULL* (*FUL*) and *SEPALLATA* 3 (*SEP3*), and inhibiting gene related to SA synthesis and SA response such as *pathogenesis-related protein 1* (*PR1*),* pathogenesis-related protein 2* (*PR2*),* isochorismate synthase 1* (*ICS1*), *allene oxide cyclase 1* (*AOC1*)*, MATE efflux family protein 5* (*EDS5*), and *plant defensin 1.2* (*PDF1.2*)^[31]^, indicating a complex cross-talk between signaling pathways of SA, UV-C light and flowering.

Flowering time-regulating stimuli varies from species to species^[32]^. Some studies have shown that the response genes of stimuli and signaling pathways downstream stimulus are not completely consistent among species^[33, 34]^. The conserved features of SA-responsive genes associated with flowering implies that SA may also promote flowering of other plants, but whether SA acts via same signaling pathways as previously identified or not needs to be further investigated.

### SA regulates pollen tube elongation during pollination

When pollen falls on the stigma, and they recognize each other, the pollen tube germinates and elongates within the stigma, eventually feeding the sperm cells into the embryo sac. Thus, pollen tube germination and elongation are important processes in plant fertilization, during which the SA content changes in plant pistils, diffusing rapidly in plant cells and regulating local pollen tube growth^[9]^.

**Figure 2:**
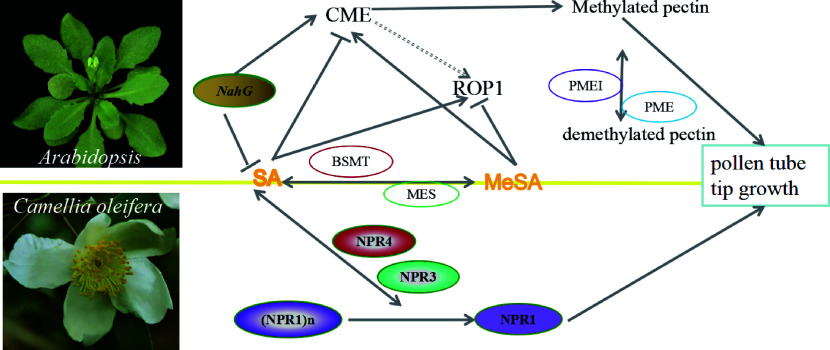
The proposed SA and MeSA models inhibit and promote pollen tube tip growth in *Arabidopsis* and *Camellia oleifera*. SA promotes pollen tube tip growth in* Arabidopsis* by regulating clathrin-mediated endocytosis, while MeSA inhibits this process. SA and MeSA have antagonistic effects on ROP activity in pollen tubes. In *C. oleifera,* *CoNPR1* and* CoNPR3.1* may be involved in SA- and MeSA-mediated pollen tube growth. CME: clathrin-mediated endocytosis; MES: Methyl salicylate esterase; PME: Pectin methyl esterase; PMEI: PME Inhibitor; BSMT: benzoic acid and SA carboxyl methyltransferase. *NahG*: *Naphthalene hydroxylase G*, which encoding salicylate hydroxylase that inactivates SA, is unable to accumulate SA, resulting in the suppression of early flowering phenotype in Arabidopsis; NPR1: Non-expressor of pathogenesis-related genes 1; NPR3: Non-expressor of pathogenesis-related genes 3; NPR4: Non-expressor of pathogenesis-related genes 4; ROP1: Rho-of-Plantssmall GTPase 1. The line with single arrow indicates induction. The line with transverse indicates repression. The line with double arrows indicates interconversion.

SA is involved in plant flowering and pollen tip growth. Exogenous SA could alleviate the damage of the spikelet differentiation, caused by heat stress during floret differentiation stage in rice, which leads to pollen reversal abortion under normal circumstances^[10, 35, 36]^. In *Arabidopsis*, SA inhibits pollen tube elongation, while methyl salicylic acid (MeSA) does the opposite. Under the action of SA methyltransferase and MeSA methylesterase, SA and MeSA are reciprocally converted to play important roles in pollen tube elongation by regulating Rho-of-Plantssmall GTPase (ROP) activity, clathrin-mediated endocytosis (CME), and cell polarity. The effects of SA and MeSA on pollen tube growth are independent of the Non-expressor of pathogenesis-related genes *3/4* (*NPR3/4*) SA receptors and Non-expressor of pathogenesis-related genes *1* (*NPR1*) which regulates nuclear gene expression^[11]^. In self-incompatibility of *Camellia oleifera,* SA content was significantly higher in self-pollinated stamens than in cross-pollinated stamens, suggesting that SA may be involved in regulating pollen tube germination and pollen tube elongation to promote the self-incompatibility process. The expression of *CoNPR1* and *CoNPR3.1* in pollen tubes were regulated by SA or MeSA, whose expression patterns were identical during the pollen germination process (Fig. 2). These results indicate that the *CoNPRs* might play an important role therein^[9]^. However, a study in pear pollen tubes showed that stamens produced SA in large amounts after pollination to promote pollen tube germination and pollen tube elongation^[12]^, indicating a positive effect of SA on pollen tube development. All these results suggest that the regulatory roles of SA in pollen tube development have not yet been comprehensively explored and need to be further elucidated.

### SA regulates ovarian development after fertilization

**Figure 3:**
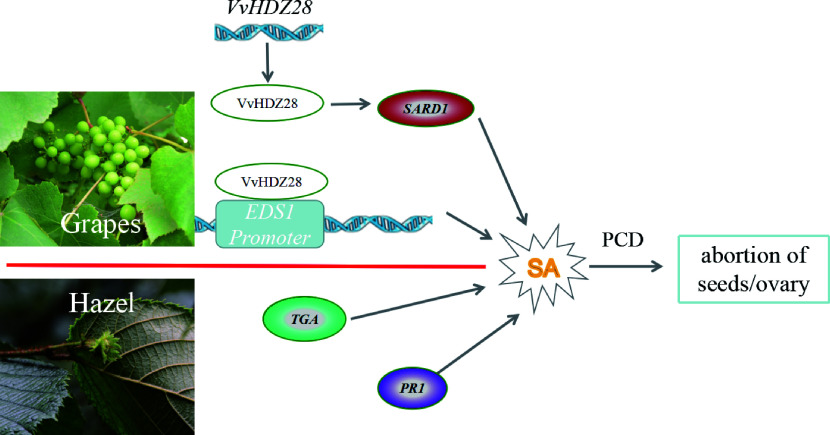
The proposed model of genetic and molecular interactions of the regulatory network during embryo abortion in grape and *Corylus spp.* (hazel) ovary abortions. In grape, VvHDZ28 up-regulates *VvEDS1* expression by binding to its promoter, and VvHDZ28 can also indirectly upregulate the positive expression of *VvSARD1*. In hazel, the SA signal transduction pathway may contribute to the regulation of abortive ovary formation via up-regulation of the TF *TGA* and pathogenesis-related protein 1 (*PR1*). The line with single arrows indicates induction. The line with transverse indicates repression. The line with double arrows indicates interconversion,* SARD1*: *Systemic acquired resistance deficient 1*; *EDS1: Enhanced disease susceptibility1*; *VvHDZ28: Homeobox domain-Zip family28* in *Vitis vinifera L..*

The development of plant embryos can be stalled at any developmental stage in response to various internal or external stresses. Moreover, SA-triggered PCD processes have also been identified in this process^[13−15]^. In* Corylus spp*., the SA precursor was significantly enriched in ovarian development and the SA signal transduction pathway may contribute to the regulation of abortive ovary formation via up-regulation of the *transcription factor TGA* (*TGA*) and pathogenesis-related protein 1 (*PR1*), indicating that high levels of SA accumulation can hinder post-fertilization ovary development^[13]^. Similar phenomena exist in seedless grape fruit development (Fig. 3), the TF of *VvHDZ28* up-regulates *VvEDS1* expression by binding to its promoter. Otherwise, VvHDZ28 can also indirectly upregulate *VvSARD1* to promote the accumulation of SA during episperm and endosperm development. And then, the accumulation of SA promotes PCD in embryonic cells and leads to abortion of grape seeds^[14, 15]^.

### Stress-induced flowering and flower development

Flowering is also induced upon in abiotic stresses and pathogen attack, and stress-induced flowering has been found in many plants where SA plays a key role^[33, 37]^.

Most of the environmental factors promoting plant flowering can be considered as stresses^[38]^. SA not only accelerates the transition from vegetative to reproductive phases in stressed plants, but also serves as a regulator of flowering time in non-stressed plants. SA can negatively regulate floral repressor genes such as *FLC* and affect the components of the autonomous flowering pathway^[25,39]^. The association between plant immunity and the flowering process has been studied in *Arabidopsis*, where pathogen attack suppresses the nuclear factors YB2 and YB3 to induce the endogenous hormones SA and Jasmonic acid (JA) which then promote flowering^[40]^. It has been speculated that stress-induced flowering increases the chances of survival of plant populations during the transitional period of stressful environments. Even if plants cannot adapt to adverse environmental conditions, this could produce the next generation as an emergency response^[33]^.

## PROSPECTS

In this paper, we reviewed the progress of studies on the role of SA in flower development over the past decades. We also described our knowledge gaps regarding the regulatory mechanisms by which SA controls flower formation. There are still many phenomena that remain unexplained in the process of flower development and morphological differentiation in plants. In summary, the study of SA in flower development physiology is poorly understood and needs to be further investigated:

1. The regulation mechanism of the SA signaling pathway in flowering is still unclear. At present, some studies have shown that SA is involved in the regulation of many reproductive processes, such as flowering, stamen failure in female flowers, pollen tube elongation and ovary development after fertilization, but few studies have manifested the specific regulatory genes working in these processes. Which flowering-related genes are SA-responsive genes? How SA regulates these flowering-related genes? Which stimuli trigger SA accumulation during flowering processes? How plants perceive SA signal during flowering and what's the receptor? What are the kinases and transcription factors participating in the flowering-regulating SA signal transduction downstream of SA receptor, and how they work? Exploring the components in the above signal pathways and their regulatory relationships as well as the cross-talk between SA-involved plant-defense and flowering processes is essential for the overall understanding of the plant SA signaling and flower development.

2. It remains unclear how SA mechanistically coordinates different biologic pathways. For example, SA in tung tree triggers tapetum PCD and stamen abortion in female flowers, but whether the mechanism underling SA-induced is almost the same for plant immunity and stamen abortion remains to be investigated. What's more, it is reported that SA promotes flowering while resisting diseases, the mechanism of how SA coordinates flowering and plant defense under certain conditions deserves further study. These studies will help to analyze complex SA signaling networks and identify their important roles in various physiological process.

3. The SA concentrations and functions could be various in different species, tissues, and in response to different development stages and environmental conditions, because of the complexity and specificity of gene expression and internal/external stimuli, which are worthy of in-depth study to gain a thorough understanding of the regulatory role of SA.

4. Florescence rate and fruit setting rate are important parameters directly related to the productivity of economic forests for yield. It is reported that both exogenous and endogenous SA have positive function on flower- and fruit-related metabolic process, inducing flowering, accelerating flower bud and pod formation, improving fruit setting rate in *L. gibba*^[29]^*, Glycine max*^[41]^ and *Abelmoschus esculentus*^[42]^. It is indicated that SA might be applied to increase the yield of economic forests in practice. However, the working mechanism, the optimal treatment concentrations and stages remain to be investigated. Overall, it would be the next-stage target for our further research of SA-involved flowering process in non-wood forests.
